# Real-world data to evaluate effects of a multi-level dissemination strategy on access, outcomes, and equity of monoclonal antibodies for COVID-19

**DOI:** 10.1017/cts.2023.679

**Published:** 2023-11-13

**Authors:** Mika K. Hamer, Chelsea Sobczak, Lindsey Whittington, Rachel L. Bowyer, Ramona Koren, Joel A. Begay, Hillary D. Lum, Adit A. Ginde, Matthew K. Wynia, Bethany M. Kwan

**Affiliations:** 1 Center for Bioethics and Humanities, University of Colorado Anschutz Medical Campus, Aurora, CO, USA; 2 Department of Family Medicine, University of Colorado Anschutz Medical Campus, Aurora, CO, USA; 3 Colorado Health Institute, Denver, CO, USA; 4 Patient Partner/Community Affiliate, University of Colorado Anschutz Medical Campus, Aurora, CO, USA; 5 Johns Hopkins Center for Indigenous Health, Department of International Health, Johns Hopkins Bloomberg School of Public Health, Baltimore, MD, USA; 6 Division of Geriatric Medicine, Department of Medicine, University of Colorado School of Medicine, Aurora, CO, USA; 7 Department of Emergency Medicine, University of Colorado School of Medicine, Aurora, CO, USA; 8 Colorado Clinical & Translational Sciences Institute, University of Colorado Anschutz Medical Campus, Aurora, CO, USA; 9 Division of General Internal Medicine, University of Colorado School of Medicine, Aurora, CO, USA

**Keywords:** COVID-19, monoclonal antibodies, dissemination and implementation, stakeholder engagement, real-world effectiveness

## Abstract

**Introduction:**

Multi-level dissemination strategies are needed to increase equitable access to effective treatment for high-risk outpatients with COVID-19, particularly among patients from disproportionately affected communities. Yet assessing population-level impact of such strategies can be challenging.

**Methods:**

In collaboration with key contributors in Colorado, we conducted a retrospective cohort study to evaluate a multi-level dissemination strategy for neutralizing monoclonal antibody (mAb) treatment. Real-world data included county-level, de-identified output from a statewide mAb referral registry linked with publicly available epidemiological data. Outcomes included weekly number of mAb referrals, unique referring clinicians, and COVID-19 hospitalization rates. We assessed weekly changes in outcomes after dissemination strategies launched in July 2021.

**Results:**

Overall, mAb referrals increased from a weekly average of 3.0 to 15.5, with an increase of 1.3 to 42.1 additional referrals per county in each post-period week (*p* < .05). Number of referring clinicians increased from a weekly average of 2.2 to 9.7, with an additional 1.5 to 22.2 unique referring clinicians observed per county per week beginning 5 weeks post-launch (*p* < .001). Larger effects were observed in communities specifically prioritized by the dissemination strategies. There were no observed differences in COVID-19 hospitalization rates between counties with and without mAb treatment sites.

**Conclusion:**

Real-world data can be used to estimate population impact of multi-level dissemination strategies. The launch of these strategies corresponded with increases in mAb referrals, but no apparent population-level effects on hospitalization outcomes. Strengths of this analytic approach include pragmatism and efficiency, whereas limitations include inability to control for other contemporaneous trends.

## Introduction

In November 2020, the US Food and Drug Administration issued an emergency use authorization (EUA) for the first evidence-based treatment for outpatients with COVID-19 – bamlanivimab, a neutralizing monoclonal antibody (mAb) therapy [1] – based on clinical trial data demonstrating efficacy in prevention of hospitalization [2]. While this was a major advance in the fight to mitigate ongoing waves of morbidity and mortality stemming from the COVID-19 pandemic, uptake of the treatment in the US was slow [3,4]. Contrary to expectations that high demand would outstrip supply, leading to the need to prioritize only the highest-risk patients for treatment, few people infected with COVID-19 were being treated as outpatients before needing to be hospitalized [4]. By spring of 2021, two additional monoclonal antibody treatments had been granted EUAs – and increasing evidence supported the effectiveness and safety of mAbs as outpatient COVID-19 treatment [5–7] - but still fewer than 5% of available doses had been used, despite widespread and ongoing infection, hospitalization, and death [8]. To address this problem, federal and state governments, public health departments, and health care systems across the US sought to rapidly enhance access to and use of mAbs for COVID-19 [4]. Yet there was little evidence – and limited infrastructure – to guide these implementation efforts. Furthermore, anticipated challenges related to equitable access to care threatened to exacerbate the already-present disparities in COVID-19 outcomes among certain racial and ethnic groups, frontline health care and other essential workers, and in rural communities [9,10].

In response to these challenges, in March 2021 the National Center for Advancing Translational Sciences funded the Colorado Clinical & Translational Sciences Institute, in part, to develop and test mAb treatment dissemination and implementation (D&I) strategies. Using methods and theories from the field of D&I science, the “mAb Colorado” team conducted surveys, interviews, and focus groups with community members and health care professionals in the state of Colorado to understand barriers and facilitators to equitable access to mAbs for COVID-19 [11– 13]. In parallel, the team engaged clinicians and community members in co-design of dissemination strategies to enhance awareness of mAb availability, referral, and treatment processes in the state (unpublished data). The multi-level dissemination strategy followed recommendations from Brownson and colleagues [14] for disseminating public health science. We applied a dissemination framework (diffusion of innovations) [15] and engaged with academic and public partners (community members, policymakers, clinicians) to develop messages and materials useful to those expected to take action [11–13]. Table 1 summarizes the audience, packaging, and communication channels, and provides example messages for each of the co-designed products. Details about the selection, development, and communication of the comprehensive dissemination strategy are reported elsewhere [16]. We launched all main components of the dissemination strategy (newsletters, website, social media, radio) in July 2021 and continued adding and iterating content through December 2021.

**Table 1.** Summary of mAb Colorado dissemination strategies

Audience Segments	Example Messages	Packaging	Communication channels
Community Members	Monoclonal antibodies (mAbs) for COVID-19 are a safe and effective treatment for people at high risk for severe disease. mAbs can stop symptoms fast and save lives. The treatment works by giving temporary enhanced immunity, which can keep you from getting sicker. Feel better faster and stay out of the hospital. If you test positive for COVID-19 and are high risk, call a medical provider or visit an urgent care center right away and get a referral for mAb treatment. mAbs work best within a few days after symptoms start. Test. Treat. Isolate. There are 30+ infusion centers in Colorado. The medication is free. Treatment is available regardless of insurance or immigration status.	Print and electronic flyers Social media postsRadio spots and social media “takeovers” Postcards Graphic Novel (static and dynamic, print and electronic) Personal stories and testimonials (All in English and Spanish)	mAb Colorado Project website Social Media (Facebook, Twitter, Instagram, YouTube) Radio Direct Mail Virtual Town Halls Live news
Clinicians	Guidance on assessing eligibility for mAbs, assessing patient interest, finding an infusion center, arranging treatment, and explaining costs. Implementation guidance for referrals, intravenous and subcutaneous treatment, and local public health processes. Example language for counseling patients on COVID-19 mAb treatment. Summaries of Emergency Use Authorizations, journal articles and press releases	One-pagers (print and electronic) Implementation blueprint “how to” guide Webinars mAb Referral and eligibility checklist	mAb Colorado Project website Email newsletters Presentations to local public health, clinics, academic partners, and professional organizations Word of mouth
Public Health, Federal, and Health System Leaders	Emerging findings on dissemination and implementation barriers, equity and access, and real-world effectiveness	Executive summaries Infographics Personal communications	Email Direct communication to key decision makers

The dissemination strategies we used included community-focused communication campaigns, clinician education and guidance materials, and capacity building through partnerships with health care systems and public health agencies. A key partner in this work was the Colorado Department of Public Health and Environment (CDPHE; the state health department), which had developed a secure, web-based referral system to help clinicians connect patients to mAb treatment sites with available doses. This state system, called “the mAb Connector Tool,” yielded a database of referrals that included patient information, referral date, referring clinician, and referral site. While primarily designed to support clinical care, this database provided a prime source of “real world data” to evaluate the impact of the mAb Colorado dissemination strategies on referral patterns and trajectories statewide.

This paper describes the real-world data methods used to assess changes in Colorado’s mAb referral rates before and after launch of the mAb Colorado multi-level dissemination strategy. In combination with publicly available data on COVID-19 infection and hospitalization rates and population demographics, we conducted a retrospective cohort study using mAb Connector Tool referral data to evaluate: 1) total mAb referrals; 2) number of unique referring clinicians; and 3) COVID-19 hospitalization rates (per 100,000 population) to assess the population-level impact of the mAb Colorado dissemination strategies in terms of behavior change (referrals and referring providers) and outcomes from having received treatment (hospitalization rates). These outcomes were selected from the available comprehensive, real-world data collected by the state health department and used to measure population-level effects. We assess the equitable impact of the mAb Colorado dissemination strategies by examining differences in the outcomes among communities specifically prioritized by these efforts. Communities experiencing disproportionately high burden imposed by COVID-19 (e.g., rural/frontier vs. urban counties, counties with high proportions of Hispanic/Latinx residents) were a focal point of the mAb Colorado dissemination strategies.

## Materials and methods

### Study design

In this retrospective cohort study of real-world data, we used mAb referral records provided by CDPHE, county COVID-19 hospitalization data, and demographic data on county population composition to address the study objectives of demonstrating the impact of the mAb Colorado dissemination strategies on mAb access, outcomes, and equity. We limited the timeframe of our analysis to November 2020–December 2021, when one or more mAbs were authorized and available for high-risk outpatients with COVID-19 in Colorado. In January 2022, the EUAs were revoked for two of three mAb products that had been available due to demonstrated lack of efficacy against the then-dominant omicron variant [17]. Low supply of sotrovimab, the remaining effective mAb with an EUA, and availability of alternative treatments meant that doses were allocated and distributed in ways that differed from most of 2021.

### Data sources

We compiled data from multiple sources and aggregated observations at the county-level each week from November 29, 2020 to December 26, 2021. Counts of mAb referrals over time came from CDPHE’s mAb Connector Tool, a HIPAA-compliant, REDCap-based form submission tool to enable clinicians to refer patients for mAb treatment. The connector tool form gathered patient-level qualifying information (mAb eligibility criteria), desired mAb treatment site, and a clinician identifier. The tool also included a list and a map of all treatment sites operating in the state (including both intravenous infusion sites and sites offering subcutaneous injection) that wished to be listed. Form submissions were automatically directed to the designated contact at the specified treatment location. This system served as a registry of referrals but did not track actual receipt of treatment. CDPHE personnel prepared a data extract for the study team consisting of weekly counts of referrals and unique referring clinicians for each treatment site. County-level hospitalization rates came from the US Department of Health and Human Services and are reported as the sum of the average number of reported patients currently hospitalized in an inpatient bed who have suspected or confirmed COVID-19 reported during the 7-day period in each hospital per county per 100,000 population [18]. COVID-19 case rates and counts came from the CDPHE COVID-19 County-Level Open Data Repository [19].

We explored multiple model specifications that included many different population-level characteristics as covariates, many of which were highly collinear. Due to small sample sizes and highly correlated covariates introducing noise or diluting/inflating the observed outcomes, the final model includes a limited number of covariates that conceptually were most likely to be associated with mAb referrals and that reflected the priority populations of the mAb Colorado dissemination strategies. These included county type (urban/rural/frontier), share of the population over age 65, by racial/ethnic group, and voting for the Republican presidential candidate in 2020. Fixed (time-invariant) county demographics came from the Colorado Department of Local Affairs, State Demography Office, and County Population Estimates (including urban/rural/frontier county designation). County voting patterns came from the 2020 Presidential Election Results from the Colorado Secretary of State [20,21].

### Intervention

Our intervention consisted of a multi-level, comprehensive dissemination strategy with materials and messages designed for community and clinician audiences. The project also supported enhanced system capacity for mAb treatment through partnerships with health systems and influencing statewide policy change. For instance, the study team advocated to the state to implement mobile buses and enable self-referral, which was implemented in September 2021. These activities were designed in response to community and clinician feedback [11–13] and in partnership with local and state entities and departments of public health. Using community engagement studio methods [22], we co-designed community-focused messages and materials for five key audiences: general community members, community members living in rural areas, community members with lower literacy, members of American Indian/Alaskan Native communities in Colorado, and Spanish-speaking community members [23–25]. These messages and materials were disseminated through the project website (www.mabcolorado.org), social media (both paid and unpaid), radio, postcards, flyers in local primary care clinics and emergency departments, and through Google search optimization. We also co-designed clinician-focused messages and materials, including a quick reference patient eligibility and referral checklist, an implementation blueprint, and PowerPoint presentations describing the strength of evidence and referral and implementation guidelines. These materials were distributed through a newsletter that reached more than 300 subscribers across the state, paper delivery to clinics by regional health connectors, and presentations to clinician audiences statewide including several well-attended webinars in partnership with ECHO Colorado [26]. Members of the mAb Colorado team conducted multiple state and national interviews with television and newspaper outlets, many of which were coordinated through the University of Colorado communication office. These dissemination activities launched in July 2021 and continued through fall of 2021.

### Setting

To evaluate the overall impact of these dissemination strategies, we define a post-period (July 2021 and later) when the mAb project activities were actively being disseminated, though not always at regular intervals. Our cohort was defined as the 23 counties in Colorado with at least one mAb treatment site documented as active during the study period. In the comparative analyses of COVID-19 hospitalization rates, the comparison group consisted of the 41 Colorado counties without a mAb treatment site at any point during the study period.

### Outcomes

We estimated models for multiple outcomes to address our study objectives: total mAb referrals, number of unique referring clinicians, and COVID-19 hospitalization rate (per 100,000 population). Total referrals and number of unique referring providers were direct indicators of the effectiveness of our dissemination strategies and are likely to have been affected by patient-, clinician-, and health-system-facing materials. Absent statewide patient-level data, we used county-level hospitalization rate as a proxy for population-level impact. Hospitalization rate is a distal measure of the effectiveness of having received treatment, one which we expect to have decreased as a result of the mAb Colorado efforts. To address questions of equity and examine communities prioritized by mAb Colorado dissemination strategies, we stratified on county and population characteristics. To examine differences by urbanicity, we stratified into urban versus rural and frontier counties combined (due to small numbers). To examine differences by race/ethnicity, we stratified on the share of the population identifying as Hispanic/Latinx, using 20.6% (the observed mean population share) as the threshold for high- versus low-Hispanic/Latinx population share.

### Model specification

We estimated separate models to evaluate the multiple outcomes of interest. To evaluate the association between mAb Colorado dissemination strategies and total mAb referrals and unique referring clinicians by week, we used zero-inflated negative binomial models due to conditional overdispersion and the large number of zeros observed for the count outcomes [27]. The zero-inflated model assumes two mechanisms, and thus two distinct groups of counties, with a zero value for the outcome. Specifically, there is one group of counties whose zero mAb referrals and referring clinicians are generated by the standard negative binomial distribution, and a second group of counties that were “always zeros” because they have zero probability of a mAb referral or a referring clinician count greater than zero; observations of zero mAb referrals or referring clinicians may come from either group. The always zeros here refer to counties with zero COVID-19 cases reported in a given week. These counties are distinct from those with COVID-19 cases but no referrals for mAbs. Zero-inflated negative binomial regression estimates the outcome in two parts that account for the generation of a zero through the two separate mechanisms. The final model specification was selected based on BIC. We favored a more parsimonious model to improve explainability and because of relatively small sample sizes.

For the comparative estimation of association between mAb Colorado dissemination strategies and COVID-19 hospitalization rates, we used a zero-inflated negative binomial model. The final model specification was selected on BIC. To allow for heterogeneous effects by time, we modeled “T” post-periods representing each week in July 2021 and later when the mAb Colorado strategies were being disseminated. The model was of the form:

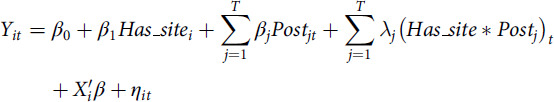




where *i* indexed county at time *t*, *Has_site* is an indicator for presence of a mAb treatment site and *Post* is an indicator for each week in the post-period from July to December 2021. The effects of the mAb Colorado dissemination strategies by time are given by the coefficients λ_j_. Thus, the treatment effect by time is a comparison of expected outcomes in each post-period *j* = 1 to T relative to the combined pre-period (November 2020–June 2021). *X’*_i_ represents a vector of time-invariant county characteristics (county urbanicity, key population demographics); *η* denotes cluster robust standard errors at the county-level. Because the coefficients from the negative binomial models are not directly interpretable, we used predictive margins to obtain the predicted number of events for the cohort and comparison groups each week. We calculate the relative effect in the cohort counties by subtracting pre- from post-estimates for each county group, then taking the difference of those values (i.e., the within- and between-group differences for each week in the post-period). We account for the observation of hospitalization to be delayed after COVID-19 infection and treatment by including a 2-week lag for this outcome.

This study was approved as non-human subjects research (all data were either publicly available or aggregated at the county and week level) by the Colorado Multiple Institutional Review Board (#21-2872). All analyses were conducted using Stata v.16 (College Station, TX). We considered statistical significance at the *p* = 0.05 level using 2-sided tests of significance.

## Results

Among the 64 Colorado counties, 23 (35.9%) had at least one mAb treatment site during the study period (Table 2). Compared to counties without a mAb treatment site, counties with a treatment site had larger populations (*p* = 0.002) and were more likely to be urban (39.1% vs. 19.5%, *p* = 0.015). Population characteristics were otherwise similar in terms of age, racial and ethnic group distribution, poverty, and socio-political context (measured by county-level share of Republican candidate votes in the 2020 presidential election) (all *p* > 0.05). The average absolute number of COVID-19 cases per week was higher in the counties with a mAb treatment site (381.9 vs. 69.9, *p* < 0.001), though the COVID-19 case rate per 100,000 people was similar (224.3 vs. 219.3, *p* = 0.65). By the end of 2021, COVID-19 vaccine series completion among people aged 12 years and older was higher in counties with a mAb treatment site (67.3% vs. 58.2%, *p* = 0.05).

**Table 2.** Characteristics of Colorado counties, overall and by presence of mAb treatment sites

	Overall	Counties with mAb treatment site(s)	Counties without mAb treatment site(s)	*P*-value
Counties (*N*)	64	23	41	
Number of sites (mean, sd)	0.627 (1.00)	1.66 (0.977)	0 (0)	
County characteristics				
Population (mean, sd)	91,112 (185,309)	185,901 (26,2931)	37,938 (89,425)	0.002
County type (*N*, %)				0.015
Urban	17 (26.6%)	9 (39.13%)	8 (19.51%)	
Rural	24 (37.5%)	11 (47.83%)	13 (31.71%)	
Frontier	23 (35.4%)	3 (13.04%)	20 (48.78%)	
Median Age (mean, sd)	42.3 (5.2)	40.1 (4.10)	43.1 (5.54)	0.10
Population racial/ethnic demographics (mean %, sd)				
White, non-Hispanic	73.4% (14.1)	71.6% (13.0)	74.4% (14.8)	0.45
Black/African American, non-Hispanic	2.04% (2.3)	2.53% (3.0)	1.77% (1.8)	0.21
Asian, non-Hispanic	1.52% (1.4)	1.79% (1.5)	1.37% (1.4)	0.27
American Indian/Alaska Native, non-Hispanic	2.34% (2.1)	2.42% (2.8)	2.29% (1.7)	0.82
Hispanic/Latinx	20.6% (13.5)	21.4% (12.0)	20.1% (14.4)	0.72
Population poverty (mean %, sd)				
Under 100% FPL	12.2% (5.2)	11.8% (4.0)	12.4% (5.7)	0.69
100–199% FPL	18.8% (6.3)	17.6% (3.9)	19.5% (7.3)	0.26
200–299% FPL	18.0% (4.1)	18.0% (3.2)	17.9% (4.6)	0.95
300–399% FPL	14.2% (3.5)	14.1% (1.8)	14.3% (4.2)	0.82
400% FPL and Up	36.8% (12.7)	38.5% (8.7)	35.9% (14.5)	0.44
Socio-Political Context (mean %, sd), Share of votes for Republican presidential candidate (2020)	56.0% (18.9)	55.3% (17.8)	56.3% (19.7)	0.85
COVID-19 demographics				
COVID cases per week (mean, sd)	187.6 (571.6)	381.9 (787.3)	69.9 (335.7)	<0.001
COVID case rate per week, per 100,000 (mean, sd)	221.2 (326.7)	224.3 (223.0)	219.3 (375.9)	0.65
Vaccine series complete by 12/31/2021, age 12+ (mean %, sd)	60.6 (16.4)	67.3 (11.7)	58.2 (17.3)	0.05

*Note:* T-tests were used to compare group means for continuous variables; Pearson *X* ^2^ tests were used to compare categorical variables. *Abbreviations:* mAb: neutralizing monoclonal antibodies; SD: standard deviation; FPL: Federal poverty level.

**Figure 1.**
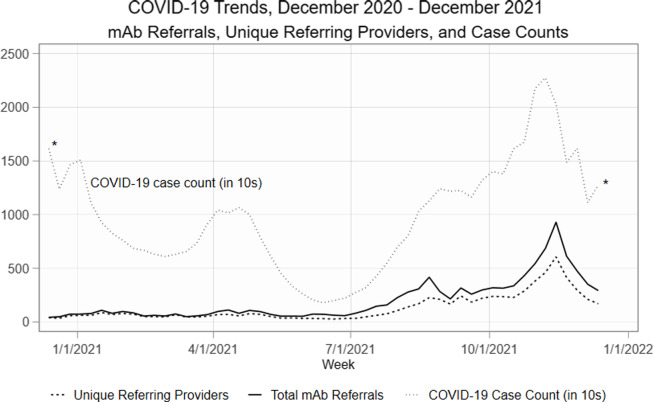
COVID-19 case counts, total mAb referrals, and unique referring providers, December 2020–December 2021. *Note:* *High case counts in November 2020 and the latter half of December 2021 distort the graph, making trends in mAb referrals and unique referring clinicians indistinguishable. For clarity of presentation, we omit those observations from Fig. 1 (though the observations are included in the analysis). *Abbreviations:* mAb = neutralizing monoclonal antibodies.

Despite the availability of mAbs and the presence of COVID-19 cases in the pre-period, there were few referrals for mAbs before July 2021 (Fig. 1). The number of referrals and unique referring clinicians increased after the mAb Colorado dissemination strategies began. In counties with at least one recorded COVID-19 case and mAb treatment site, the weekly average number of mAb referrals increased from 2.99 in the pre-period from November 29, 2020 to June 30, 2021 to 15.47 in the post-period from July 1 to December 30, 2021, a 417.4 percentage point increase. Number of unique referring clinicians increased from a weekly average of 2.20 in the pre-period to 9.73 in the post-period, a 342.3 percentage point increase.

**Figure 2.**
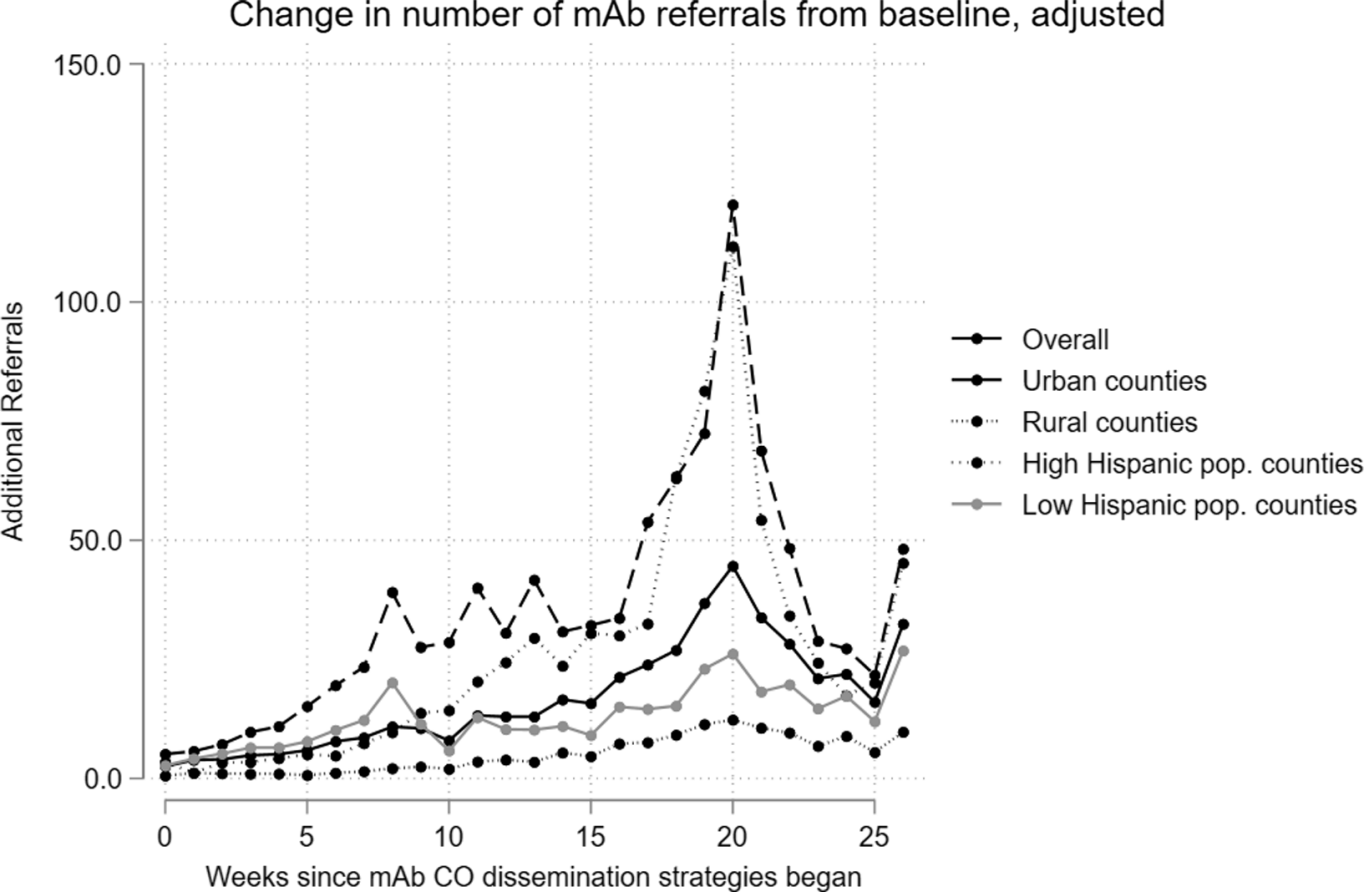
Adjusted change in mAb referral counts from baseline (Nov 2020–June 2021), overall and by county type.

**Figure 3.**
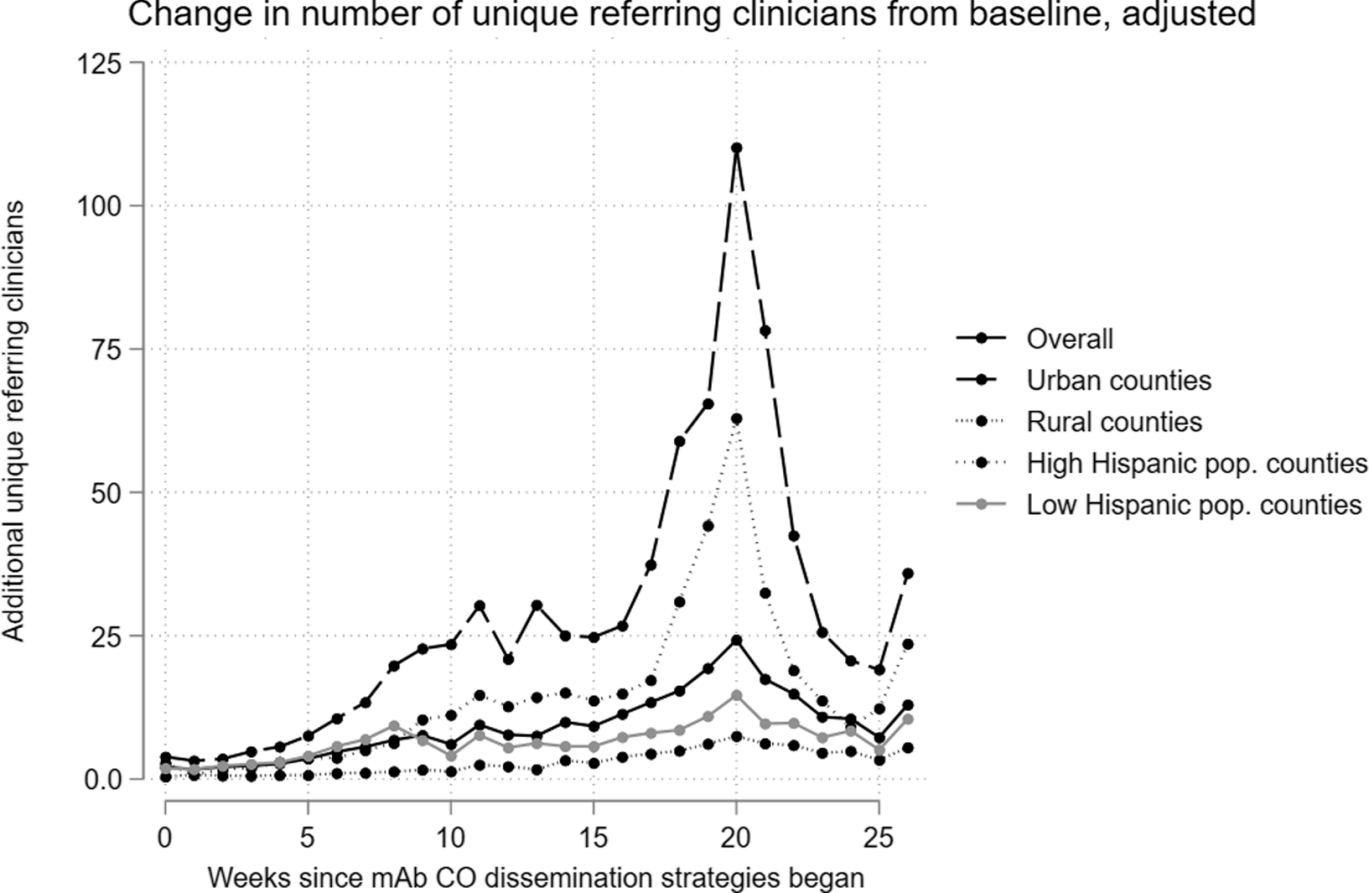
Adjusted change in unique referring clinicians from baseline (Nov 2020–June 2021), overall and by county type.

Marginal effects from the zero-inflated negative binomial regression models for the mAb referral and unique referring clinician outcomes are displayed in Figs. 2 and 3 (full regression output reported in Supplemental Table S1 and Table S2). There was a statistically significant increase in the number of mAb referrals each week from July 4 to December 26, 2021, compared to the pre-period average. The magnitude of the increase was variable by time, ranging between 1 and 42 additional mAb referrals per county per week in the post-period (all *p* < 0.05). Compared to urban counties, there were approximately 10 fewer mAb referrals per week in rural and frontier counties.

The number of unique referring clinicians increased over the pre-period average beginning in August 2021, approximately five weeks after the mAb Colorado dissemination strategies launched (*p* < 0.001). There was a variable increase in the number of unique referring clinicians, ranging from 1 to 22 additional clinicians per county per week. Like mAb referrals, there were approximately 6 fewer unique referring clinicians per rural and frontier county per week, compared to urban counties.

In stratified models by urban and rural/frontier designation (rural and frontier combined due to a small number of frontier counties), trends were similar (Fig. 2, Supplemental Table S1). However, the increase in mAb referrals was much greater in urban counties. Population demographics contributed differentially in urban versus rural/frontier counties. In urban counties, a lower number of mAb referrals was observed when there were higher percentages of the population over age 65 (15 fewer referrals for every 1% increase in population over 65, *p* < 0.001) and higher percentages of Hispanic/Latinx (8.5 fewer mAb referrals per 1% increase, *p* < 0.001) and Black or African American (5.9 fewer mAb referrals for every 1% increase, *p* < 0.001) residents. Increased population size of American Indian/ Alaskan Native individuals was associated with more mAb referrals (124.6 more referrals for every 1% increase in share of total population, *p* < 0.001). There were no corresponding trends in mAb referrals according to population demographics in the rural and frontier counties, which tended to be less racially/ethnically diverse than urban counties. Similar patterns were observed, but to a lesser extent, for the number of unique referring clinicians outcome (Fig. 3, Supplemental Table S2).

In additional models, we stratified by high and low-Hispanic/Latinx population to assess impact on this community, which was an additional priority of the mAb Colorado dissemination strategy (Fig. 2, Supplemental Table S1). There were earlier increases in mAb referrals among low-Hispanic/Latinx population counties (beginning in week 1 post-launch), though counties with higher Hispanic/Latinx populations saw increases in mAb referrals of greater magnitude as time progressed. Rural and frontier counties with low-Hispanic/Latinx populations saw fewer mAb referrals than their urban counterparts (*p* < 0.05). Further, counties with larger Hispanic/Latinx shares of the population also saw greater mAb referrals as their population share of Black or African American (3.25 more referrals for every 1% increase in population share, *p* < 0.05) and American Indian/Alaskan Native increased (12.88 more referrals for every 1% increase in population share, *p* < 0.001). There were few population demographics associated with unique referring clinicians when stratifying by Hispanic/Latinx population share (Fig. 3, Supplemental Table S2).

**Figure 4.**
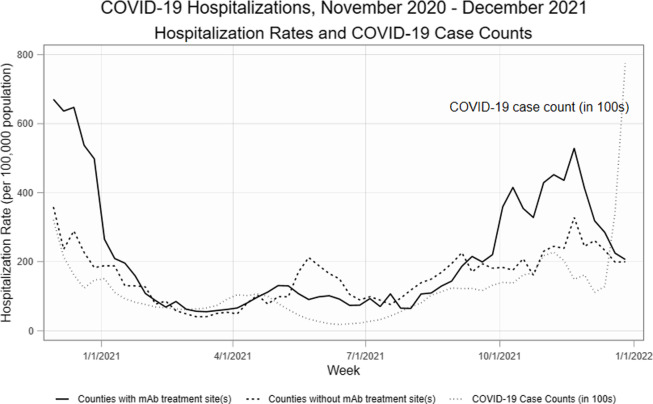
COVID-19 case counts and COVID-19 hospitalization rates by county presence of mAb treatment sites, November 2020–December 2021.

**Figure 5.**
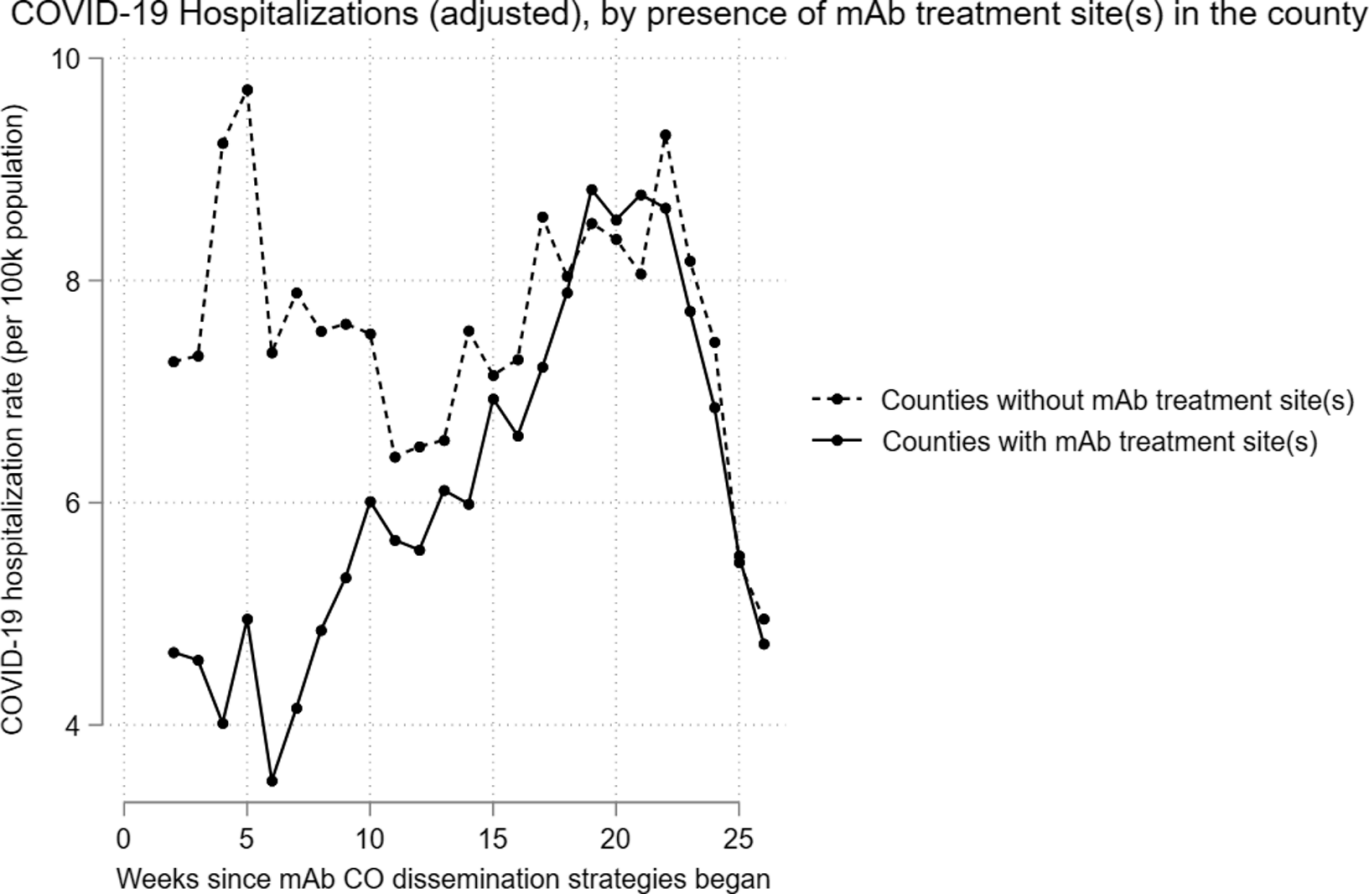
Adjusted COVID-19 hospitalization rates, by presence of mAb treatment site(s).

Hospitalization rates were variable over our study period, slightly lagging the trends in COVID-19 cases (Fig. 4). There were no sustained statistically significant differences in COVID-19 hospitalization rates between counties with and without mAb treatment sites (Fig. 5, Supplemental Table S3) after the mAb Colorado dissemination strategies were launched.

## Discussion

Using real-world data, our analyses suggest that launching the mAb Colorado multi-level dissemination strategy (community-, health system-, and public health policy level) was associated with a significant increase in mAb referrals and number of referring clinicians in counties with available mAb treatment sites. Urban American Indian/Alaskan Native populations and communities with higher proportions of historically underrepresented racial and ethnic minority residents (especially Hispanic/Latinx) were priority communities for the mAb Colorado dissemination efforts.

There are both strengths and limitations to conducting a retrospective, observational study using real-world data. While the data suggest an increase in referrals corresponding with the mAb dissemination strategies, several other co-occurring events may have influenced the uptake of mAbs. In spring and early summer of 2021, there was optimism surrounding the freedom afforded by vaccination; case counts were lower. Other data from our study showed clinicians generally saw few, if any patients, with COVID-19 at that time [12]. Yet in late July and early August 2021, there was a new wave of COVID-19 cases stemming from the Delta variant and an increase in vaccine “breakthrough” cases, so overall demand for treatment may have been higher. However, the effects of the dissemination strategy were observed even when adjusting for case counts. Other factors might have included increased national media coverage from other states (e.g., Florida, Texas) where mAbs were being promoted aggressively [28,29]. Yet there was also media coverage of nationally-known politicians who received mAb treatment in the fall and winter of 2020, without the correspondingly high demand when mAbs first became authorized and when vaccines were still not widely available. Availability of mAbs was influenced by changes in the federal mAb allocation policies, such that mAbs were at first allocated by the federal government (Administration for Strategic Preparedness and Response) to the state health departments in limited supply for distribution to healthcare facilities (November 2020 to February 2021), then loosened to allow treatment sites to order product directly from the federal distributor (February 2021 to September 2021), then returned to a more restrictive federal allocation strategy (September 2021 to December 2021) [30].

Even with increased referrals for mAbs, the results did not show a corresponding decrease in COVID-19 hospitalizations after implementing the dissemination strategies. Estimates from this analysis indicate a maximum of about 30–40 additional mAb referrals per week in counties with mAb treatment sites following the launch of dissemination strategies; other real-world effectiveness evidence from the time showed a number needed to treat of 20–30 for mAbs to avert 1 hospitalization and approximately 100 to prevent 1 death [31]. Thus, it follows that the increase in mAb referrals after the launch of mAb Colorado dissemination strategies induced no observable difference in hospitalization rates. It is also the case that our data only reports on referrals, rather than actual receipt of treatment. It is not known how many patients with referrals were ultimately able to schedule an appointment and receive treatment promptly to alter their disease course, nor how many may have desired treatment but were unable to access it due to supply shortages. In future pandemic or other large-scale public health emergency scenarios, the importance of early and consistent data collection cannot be understated. Disparate data sources greatly inhibit real-time assessment of communities most affected and make coordinated efforts to direct resources where they are most needed nearly impossible.

This study has multiple limitations. Our unit of analysis was the county, with mAb referrals being attributed to the county of treatment/referral, not patient residence. Similarly, not all counties contain a hospital, and patients may travel or be transferred outside their county of residence for the hospital admission. Benefits of mAbs would have been realized in the county of residence, not the county of referral or treatment, meaning that analysis of hospitalization outcomes would be biased toward the null. Thus, we view our findings as conservative estimates of the impact of the mAb Colorado efforts. We are not able to infer causation; while we control for a limited set of county demographics, and there is the possibility of unmeasured or residual confounders that we could not account for. We could not capture heterogeneous effects or more nuanced findings in counties where mAb Colorado dissemination efforts were especially concentrated. Using aggregate referral data, we were unable to capture individual clinician rates (per COVID-19 patient) of mAb referral. We could not distinguish between the possibility of a smaller number of high-volume “mAb referrers” who were central to increasing mAb referrals, or whether increased referrals came from more clinicians referring at similar volumes. We were not able to control for the number of mAb doses available to people in Colorado each week, so we were limited to evaluating total mAb referrals and unable to discern how much of the total state mAb supply this accounted for. Finally, there was little variability in population share of some underrepresented racial and ethnic minority groups to stratify into categories other than those presented in the paper.

In conclusion, our approach represents a pragmatic, efficient strategy to estimate the population impact of a multi-level dissemination strategy, focusing on access to treatment, outcomes, and equity of mAbs for COVID-19. In doing so, we overcame known challenges to evaluating dissemination strategies [14], which can be limited to assessment of changes in knowledge, intentions to use, or observation of policy change. While descriptive measures of the number and type of materials distributed, the number of website hits, or social media reactions can be useful, these dissemination measures do not reflect health impact or behavior change. By contrast, our use of real-world data collected by the state health department allowed for a population-level assessment of patient and clinician behavior change (referrals, referring clinicians) and patient outcomes (hospitalization rates) resulting from the multi-channel dissemination strategy. Finally, conducting randomized trials of dissemination strategies with primary data collection can be both cost and time prohibitive, and may not always be feasible or ethical [32] – especially within the context of a rapidly changing pandemic. In this analysis, we demonstrated the use of aggregate real-world data made available by the state health department to evaluate a multi-level dissemination strategy for enhancing equitable access to treatment for COVID-19. While real-world data have been used to assess ongoing mAb treatment effectiveness for COVID-19 [31,33], use to evaluate dissemination impact is innovative. We demonstrated change in two key data points – number of mAb referrals and number of unique referring clinicians – reflecting uptake and adoption of mAb referrals, especially in prioritized populations. Even as the COVID-19 pandemic transitions into an endemic phase, there are other outpatient COVID-19 therapeutics, proposed need for annual COVID-19 vaccine boosters, and future rapidly emerging public health needs that will benefit from equitable, multi-level dissemination strategies to communicate availability and access to effective prevention or treatment approaches for patients who would benefit (and the clinicians who care for them).

## Supporting information

Hamer et al. supplementary material Hamer et al. supplementary material

## Supplementary material

The supplementary material for this article can be found at https://doi.org/10.1017/cts.2023.679.

## References

[1] Mahase E. Covid-19: FDA authorises neutralising antibody bamlanivimab for non-admitted patients. BMJ 2020;371:m4362. 10.1136/bmj.m4362 33177042

[2] Chen P , Nirula A , Heller B , et al. SARS-CoV-2 neutralizing antibody LY-CoV555 in outpatients with Covid-19. N Engl J Med. 2021;384(3):229–237. 33113295 10.1056/NEJMoa2029849 PMC7646625

[3] Toy S , Walker J , Evans M. Highly touted monoclonal antibody therapies sit unused in hospitals. Wall Street Journal. 2020, 12–27. https://www.wsj.com/articles/highly-touted-monoclonal-antibody-therapies-sit-unused-in-hospitals-11609087364

[4] National Academies of Sciences Engineering Medicine. *Rapid Expert* Consultation on Allocating COVID-19 Monoclonal Antibody Therapies and Other Novel Therapeutics (January 29, 2021), 2021:34. https://www.nap.edu/catalog/26063/rapid-expert-consultation-on-allocating-covid-19-monoclonal-antibody-therapies-and-other-novel-therapeutics-january-29-2021

[5] Weinreich DM , Sivapalasingam S , Norton T , et al. REGN-COV2, a neutralizing antibody cocktail, in outpatients with Covid-19. N Engl J Med. 2021;384(3):238–251. 33332778 10.1056/NEJMoa2035002 PMC7781102

[6] Gupta A , Gonzalez-Rojas Y , Juarez E , et al. Early treatment for Covid-19 with SARS-CoV-2 neutralizing antibody sotrovimab. N Engl J Med. 2021;385(21):1941–1950. 34706189 10.1056/NEJMoa2107934

[7] Dougan M , Nirula A , Azizad M , et al. Bamlanivimab plus etesevimab in mild or moderate Covid-19. N Engl J Med. 2021;385(15):1382–1392. 34260849 10.1056/NEJMoa2102685 PMC8314785

[8] Behr CL , Maddox KEJ , Meara E , Epstein AM , Orav EJ , Barnett ML. Anti-SARS-CoV-2 monoclonal antibody distribution to high-risk medicare beneficiaries, 2020-2021. JAMA. 2022;327(10):980–983. 35119452 10.1001/jama.2022.1243 PMC8904305

[9] Wiltz JL , Feehan AK , Molinari NM , et al. Racial and ethnic disparities in receipt of medications for treatment of COVID-19—United States, March 2020-August 2021. Morb Mort Week Rep. 2022;71(3):96–102. 10.15585/mmwr.mm7103e1 PMC8774154 35051133

[10] Greene K , Huber K , D’Ambrosio M , Thoumi A , McClellan M , Plescia M , Baggett J . Maximizing the benefit of COVID-19 therapeutics: considerations for state public health officials. ASTHO and Duke Margolis Center for Health Policy Brief. 2022. https://www.astho.org/topic/brief/maximizing-benefit-of-covid-19-therapeutics-considerations-for-state-ph-officials/. Accessed September 9, 2022.

[11] Hamer MK , Alasmar A , Kwan BM , Wynia MK , Ginde AA , DeCamp MW. Referrals, access, and equity of monoclonal antibodies for outpatient COVID-19: a qualitative study of clinician perspectives. Medicine. 2022;101(50):e32191. 36550877 10.1097/MD.0000000000032191 PMC9771255

[12] Kwan BM , Sobczak C , Beaty L , et al. Clinician perspectives on monoclonal antibody treatment for high-risk outpatients with COVID-19: implications for implementation and equitable access. J Gen Intern Med. 2022;37(13):1–9. 10.1007/s11606-022-07702-2 PMC9255528 35790666

[13] Kwan BM , Sobczak C , Gorman C , et al. All of the things to everyone everywhere”: a mixed methods analysis of community perspectives on equitable access to monoclonal antibody treatment for COVID-19. PloS One. 2022;17(11):e0274043. 36417457 10.1371/journal.pone.0274043 PMC9683597

[14] Brownson RC , Eyler AA , Harris JK , Moore JB , Tabak RG. Getting the word out: new approaches for disseminating public health science. J Publ Health Manag Pract. 2018;24(2):102–111. 10.1097/PHH.0000000000000673 PMC5794246 28885319

[15] Rogers EM , Singhal A , Quinlan MM. Diffusion of Innovations. An Integrated Approach to Communication Theory and Research. Routledge; 2014:432–448.

[16] Kwan BM , Sobczak C , Begay J , et al. 2023. Rapid methods for multi-level dissemination of neutralizing monoclonal antibody treatment for COVID-19 outpatients: Designing for dissemination using the fit to context framework. Manuscript Under Review. 10.3389/fpubh.2024.1412947 PMC11617208 39639913

[17] Aggarwal NR , Beaty LE , Bennett TD , et al. Change in effectiveness of sotrovimab for preventing hospitalization and mortality for at-risk COVID-19 outpatients during an Omicron BA. 1 and BA. 1.1-predominant phase. Int J Infect Dis. 2022;128:310–317. 36229005 10.1016/j.ijid.2022.10.002 PMC9549713

[18] U.S. Department of Health & Human Services. COVID-19 reported patient impact and hospital capacity by facility. https://healthdata.gov/Hospital/COVID-19-Reported-Patient-Impact-and-Hospital-Capa/anag-cw7u. Accessed November 1, 2022.

[19] Colorado Department of Public Health and Environment. COVID19 county-level open data repository. https://data-cdphe.opendata.arcgis.com/datasets/CDPHE::cdphe-covid19-county-level-open-data-repository/explore. Accessed September 22, 2022.

[20] Colorado Secretary of State. Election results & data. https://www.sos.state.co.us/pubs/elections/resultsData.html. Accessed October 13, 2022.

[21] Sehgal NJ , Yue D , Pope E , Wang RH , Roby DH. The association between COVID-19 mortality and the county-level partisan divide in the United States: study examines the association between COVID-19 mortality and county-level political party affiliation. Health Affairs. 2022;41(6):853–863.

[22] Joosten YA , Israel TL , Williams NA , et al. Community engagement studios: a structured approach to obtaining meaningful input from stakeholders to inform research. Acad Med. 2015;90(12):1646–1650. 26107879 10.1097/ACM.0000000000000794 PMC4654264

[23] Lin Q , Paykin S , Halpern D , Martinez-Cardoso A , Kolak M. Assessment of structural barriers and racial group disparities of COVID-19 mortality with spatial analysis. JAMA Netw Open. 2022;5(3):e220984–e220984. 35244703 10.1001/jamanetworkopen.2022.0984 PMC8897755

[24] Mackey K , Ayers CK , Kondo KK , et al. Racial and ethnic disparities in COVID-19-related infections, hospitalizations, and deaths: a systematic review. Ann Intern Med. 2021;174(3):362–373. 33253040 10.7326/M20-6306 PMC7772883

[25] Raine S , Liu A , Mintz J , Wahood W , Huntley K , Haffizulla F. Racial and ethnic disparities in COVID-19 outcomes: social determination of health. Int J Environ Res Publ Health. 2020;17(21):8115. 10.3390/ijerph17218115 PMC7663309 33153162

[26] ECHO Colorado. A provider’s guide to monoclonal antibody therapy for COVID-19 Webinar. 2021. https://echocolorado.org/echo/a-providers-guide-to-monoclonal-antibody-therapy-for-covid-19/. Accessed December 6, 2021.

[27] Hardin JW , Hilbe JM. Regression models for count data based on the negative binomial (p) distribution. Stata J. 2014;14(2):280–291.

[28] Office of the Florida Governor: Ron DeSantis. Governor Ron DeSantis highlights monoclonal antibody treatment success in Florida. 2021. https://www.flgov.com/2021/10/14/governor-ron-desantis-highlights-monoclonal-antibody-treatment-success-in-florida/. Accessed February 9, 2023.

[29] Office of the Texas Governor: Greg Abbott. Office of The Governor Statement on COVID-19 monoclonal antibody treatment. 2021. https://gov.texas.gov/news/post/office-of-the-governor-statement-on-covid-19-monoclonal-antibody-treatment. Accessed February 9, 2023.

[30] American Hospital Association. HHS reinstates original distribution method for COVID-19 monoclonal antibody therapies. 2021. https://www.aha.org/special-bulletin/2021-09-17-hhs-reinstates-original-distribution-method-covid-19-monoclonal. Accessed January 15, 2023.

[31] Wynia MK , Beaty LE , Bennett TD , et al. Real-world evidence of neutralizing monoclonal antibodies for preventing hospitalization and mortality in COVID-19 outpatients. Chest. 2022;163(5):1061–1070. 36441040 10.1016/j.chest.2022.10.020 PMC9613796

[32] Mazzucca S , Tabak RG , Pilar M , et al. Variation in research designs used to test the effectiveness of dissemination and implementation strategies: a review. Front Publ Health. 2018;6:32. 10.3389/fpubh.2018.00032 PMC5826311 29515989

[33] Aggarwal NR , Beaty LE , Bennett TD , et al. Real-world evidence of the neutralizing monoclonal antibody sotrovimab for preventing hospitalization and mortality in COVID-19 outpatients. J Infect Dis. 2022;226(12):2129–2136. 35576581 10.1093/infdis/jiac206 PMC10205600

