# Supplementary material for: Real-world data to evaluate effects of a multi-level dissemination strategy on access, outcomes, and equity of monoclonal antibodies for COVID-19
Source: J Clin Transl Sci. 2023 Nov 13;7(1):e258. doi: 10.1017/cts.2023.679 (PMC10789982; doi:10.1017/cts.2023.679)
Supplement: Hamer et al. supplementary material [file S2059866123006799sup001.docx]

**SUPPLEMENTAL MATERIALS**

**Table S1.** Changes in mAb referrals according to time and county characteristics, July 1, 2021 – December 31, 2021

|  | **Overall: Marginal Effect (SE)** | **Urban Counties:**  **Marginal Effect (SE)** | **Rural/Frontier Counties:**  **Marginal Effect (SE)** | **Counties with High Hispanic/Latinx Populations:**  **Marginal Effect (SE)** | **Counties with Low Hispanic/Latinx Populations:**  **Marginal Effect (SE)** |
| --- | --- | --- | --- | --- | --- |
| **N** | 1374 | 553 | 821 | 563 | 811 |
| **Weeks Since July 1, 2021** |  |  |  |  |  |
| Nov 2020 – June 2021 | [Ref.] | [Ref.] | [Ref.] | [Ref.] | [Ref.] |
| 1 | 1.34** (0.82) | 0.60 (1.49) | 0.56** (0.38) | -2.23** (0.669) | 1.46* (1.02) |
| 2 | 1.48** (0.96) | 2.04 (2.19) | 0.47** (0.33) | -0.10 (1.57) | 2.50*** (1.56) |
| 3 | 2.33** (1.09) | 4.62** (2.51) | 0.38 (0.29) | -0.01 (1.51) | 3.77*** (1.58) |
| 4 | 2.57*** (1.09) | 5.80** (2.42) | 0.40* (0.24) | 0.79 (1.50) | 3.80*** (1.34) |
| 5 | 3.39*** (1.66) | 10.03*** (3.75) | 0.10 (0.14) | 1.60 (1.59) | 5.05*** (2.52) |
| 6 | 5.23*** (2.15) | 14.41*** (4.67) | 0.58** (0.30) | 1.34 (1.49) | 7.44*** (3.00) |
| 7 | 6.02*** (2.09) | 18.27*** (4.02) | 0.91** (0.34) | 3.95** (2.42) | 9.53*** (3.16) |
| 8 | 8.34*** (2.55) | 34.00*** (15.87) | 1.54*** (0.69) | 6.25** (4.15) | 17.40*** (8.95) |
| 9 | 7.99*** (2.05) | 22.44*** (10.15) | 1.88*** (0.78) | 10.31*** (4.15) | 8.75** (5.33) |
| 10 | 5.40*** (1.73) | 23.50*** (10.57) | 1.42*** (0.61) | 10.86*** (4.40) | 3.14* (2.15) |
| 11 | 10.69*** (2.43) | 34.90*** (14.43) | 2.94*** (0.81) | 16.91*** (4.53) | 10.11** (5.27) |
| 12 | 10.43*** (2.78) | 25.43*** (11.07) | 3.33*** (0.98) | 20.94*** (6.61) | 7.62** (5.17) |
| 13 | 10.43*** (2.76) | 36.57*** (16.94) | 2.89*** (0.96) | 26.07*** (11.07) | 7.53** (4.39) |
| 14 | 14.02*** (3.78) | 25.71*** (12.64) | 4.83*** (1.34) | 20.19*** (8.00) | 8.28** (5.05) |
| 15 | 13.23*** (3.47) | 27.05*** (13.25) | 4.07*** (1.05) | 27.06*** (11.35) | 6.38* (4.47) |
| 16 | 18.73*** (5.24) | 28.53*** (14.57) | 6.72*** (2.04) | 26.58*** (10.77) | 12.35** (7.63) |
| 17 | 21.35*** (6.12) | 48.73*** (23.74) | 6.99*** (2.57) | 29.06*** (10.38) | 11.86** (6.89) |
| 18 | 24.41*** (8.59) | 57.86*** (26.95) | 8.56*** (3.11) | 59.97*** (33.99) | 12.52** (6.10) |
| 19 | 34.26*** (8.90) | 67.32*** (30.66) | 10.80*** (3.17) | 77.92*** (50.36) | 20.29** (11.66) |
| 20 | 42.06*** (11.24) | 115.34*** (59.08) | 11.73*** (3.78) | 108.26*** (91.25) | 23.46*** (11.42) |
| 21 | 31.27*** (9.47) | 63.71*** (32.55) | 10.02*** (3.42) | 50.83*** (25.26) | 15.51** (9.78) |
| 22 | 25.69*** (6.72) | 43.24*** (20.27) | 9.01*** (2.98) | 30.75*** (13.39) | 17.00*** (9.25) |
| 23 | 18.44*** (5.07) | 23.72*** (9.86) | 6.21*** (2.01) | 20.88*** (9.73) | 11.95** (6.90) |
| 24 | 19.37*** (5.32) | 22.16*** (8.86) | 8.30*** (2.79) | 13.99*** (4.81) | 14.59** (8.12) |
| 25 | 13.49*** (3.03) | 16.60*** (5.50) | 4.93*** (1.26) | 16.66*** (5.90) | 9.27** (6.10) |
| 26 | 29.90*** (8.05) | 43.06*** (15.57) | 9.18*** (3.00) | 41.79*** (17.82) | 24.11*** (14.88) |
| **County Demographics** |  |  |  |  |  |
| County type |  |  |  |  |  |
| Urban | [Ref.] | -- | -- | [Ref.] | [Ref.] |
| Frontier | -10.78* (5.19) | -- | -- | 119.57** (139.18) | -15.20*** (7.20) |
| Rural | -10.01** (5.09) | -- | -- | -1.91 (4.94) | -11.80** (6.90) |
| Over age 65, population % | -0.42 (0.57) | -15.19*** (5.93) | 0.09 (0.15) | -6.14*** (3.28) | -0.80 (0.88) |
| Race/Ethnicity, population % |  |  |  |  |  |
| Hispanic/Latinx | -0.03 (0.16) | -8.53*** (3.32) | 0.03 (0.06) | -- | -- |
| Black, non-Hispanic | -0.29 (1.0) | -5.86*** (2.25) | -0.43 (0.81) | 3.25** (1.56) | -1.80* (1.37) |
| AIAN | -0.33** (0.46) | 124.59*** (48.49) | -0.18 (0.14) | 12.88*** (6.83) | -0.34 (0.32) |
| Voter Share for Republican presidential candidate (2020), population % | 0.012* (0.01) | -1.17*** (0.38) | -0.07 (0.05) | 0.20 (0.21) | 0.04 (0.20) |

*Note:* Results displayed as marginal effects (incidence rates). The standard errors are clustered at the county-level. Robust standard errors are in parentheses. **p*<0.1; ***p*<0.05; ****p*<0.001

*Abbreviations:* mAb: neutralizing monoclonal antibodies; SE: standard error; AIAN: American Indian/Alaskan Native;

**Table S2.** Change in unique referring clinicians according to time and county characteristics, July 1, 2021 – December 31, 2021

|  | **Overall: Marginal Effect (SE)** | **Urban Counties:**  **Marginal Effect (SE)** | **Rural/Frontier Counties:**  **Marginal Effect (SE)** | **Counties with High Hispanic/Latinx Populations:**  **Marginal Effect (SE)** | **Counties with Low Hispanic/Latinx Populations:**  **Marginal Effect (SE)** |
| --- | --- | --- | --- | --- | --- |
| **N** | 1374 | 553 | 821 | 563 | 811 |
| **Weeks Since July 1, 2021** |  |  |  |  |  |
| Nov 2020 – June 2021 | [Ref.] | [Ref.] | [Ref.] | [Ref.] | [Ref.] |
| 1 | -0.38 (0.69) | -0.69 (2.02) | 0.34* (0.26) | -2.06*** (0.33) | -0.15 (0.81) |
| 2 | 0.06 (0.45) | -0.35 (1.26) | 0.17 (0.17) | -1.07 (0.95) | 0.43 (0.44) |
| 3 | 0.27 (0.34) | 0.94 (2.25) | 0.12 (0.12) | -0.83 (0.53) | 0.75** (0.27) |
| 4 | 0.60 (0.38) | 1.76 (2.22) | 0.26** (0.14) | 0.17 (0.85) | 1.03** (0.37) |
| 5 | 1.47*** (0.55) | 3.68* (2.57) | 0.26** (0.12) | 0.90 (0.73) | 2.18*** (0.72) |
| 6 | 2.71*** (0.92) | 6.67** (3.68) | 0.61*** (0.28) | 0.92 (0.86) | 3.85*** (1.33) |
| 7 | 3.55*** (1.15) | 9.49** (4.44) | 0.66** (0.29) | 2.25* (1.58) | 5.04*** (1.56) |
| 8 | 4.74***(1.51) | 15.91** (9.87) | 0.89** (0.39) | 3.50 (3.28) | 7.44*** (2.78) |
| 9 | 5.57*** (1.40) | 18.86*** (8.24) | 1.19*** (0.45) | 7.60** (5.03) | 4.90*** (2.03) |
| 10 | 4.01*** (1.41) | 19.62*** (10.53) | 0.91*** (0.39) | 8.40*** (3.77) | 2.20* (1.18) |
| 11 | 7.40*** (1.82) | 26.39*** (13.31) | 2.07*** (0.56) | 11.90*** (4.42) | 5.80** (2.17) |
| 12 | 5.68*** (1.69) | 17.05*** (8.46) | 1.78*** (0.63) | 9.89*** (3.33) | 3.62** (1.98) |
| 13 | 5.46*** (1.61) | 26.48*** (14.21) | 1.27*** (0.43) | 11.50*** (4.11) | 4.37** (1.86) |
| 14 | 7.85*** (2.59) | 21.14** (12.68) | 2.84*** (0.85) | 12.29*** (4.73) | 3.85** (2.02) |
| 15 | 7.15*** (2.25) | 20.88** (12.35) | 2.38*** (0.76) | 10.92*** (3.76) | 3.83* (2.42) |
| 16 | 9.27*** (3.03) | 22.87** (15.10) | 3.46*** (1.08) | 12.13*** (4.16) | 5.44** (3.00) |
| 17 | 11.31*** (3.80) | 33.51** (21.90) | 4.00*** (1.43) | 14.46*** (5.88) | 6.16** (3.12) |
| 18 | 13.35*** (4.61) | 55.06*** (37.43) | 4.51*** (1.73) | 28.17*** (14.54) | 6.68** (3.10) |
| 19 | 17.26*** (4.90) | 61.59*** (45.73) | 5.73*** (1.90) | 41.42*** (27.08) | 9.09** (4.99) |
| 20 | 22.21*** (5.88) | 106.25*** (83.72) | 7.08*** (2.38) | 60.17** (48.80) | 12.78** (5.88) |
| 21 | 15.36*** (5.67) | 74.39*** (52.42) | 5.80*** (2.08) | 29.72*** (13.79) | 7.80** (5.03) |
| 22 | 12.80*** (4.22) | 38.59*** (24.37) | 5.50*** (1.73) | 16.18*** (6.91) | 7.89** (4.38) |
| 23 | 8.78*** (3.09) | 21.75** (13.15) | 4.15*** (1.39) | 10.91*** (4.50) | 5.39** (3.49) |
| 24 | 8.44*** (2.93) | 16.77** (11.12) | 4.47*** (1.50) | 6.25*** (2.72) | 6.55** (3.65) |
| 25 | 5.20*** (1.87) | 15.22*** (7.18) | 2.92*** (0.68) | 9.54** (6.59) | 3.25* (2.33) |
| 26 | 10.90*** (2.91) | 32.03*** (12.43) | 5.10*** (1.09) | 20.82** (18.13) | 8.62** (4.32) |
| **County Demographics** |  |  |  |  |  |
| County type |  |  |  |  |  |
| Urban | [Ref.] | -- | -- | [Ref.] | [Ref.] |
| Frontier | -6.97*** (2.15) | -- | -- | 46.76** (57.50) | -8.10*** (2.54) |
| Rural | -6.37*** (2.04) | -- | -- | -2.50 (5.26) | -6.25** (2.53) |
| Over age 65, population % | -0.10 (0.29) | -12.48*** (7.43) | 0.03 (0.09) | -3.34** (1.66) | -0.43  (0.31) |
| Race/Ethnicity, population % |  |  |  |  |  |
| Hispanic | 0.01 (0.06) | -6.81** (4.16) | 0.003 (0.03) | -- | -- |
| Black, non-Hispanic | -0.23 (0.46) | -4.05*** (1.92) | -0.05 (0.47) | 2.52 (2.56) | -0.99*** (0.42) |
| AIAN | -0.08 (0.20) | 98.80** (60.21) | -0.04 (0.07) | 8.31 (5.61) | -0.06 (0.13) |
| Voter Share for Republican presidential candidate (2020), population % | -0.17** (0.07) | -0.85*** (0.38) | -0.04 (0.03) | 0.18 (0.51) | 0.01 (0.07) |

*Note:* Results displayed as marginal effects (incidence rates). The standard errors are clustered at the county-level. Robust standard errors are in parentheses. **p*<0.1; ***p*<0.05; ****p*<0.001

*Abbreviations:* mAb: neutralizing monoclonal antibodies; SE: standard error; AIAN: American Indian/Alaskan Native;

**Table S3.** COVID-19 hospitalizations, displayed as hospitalizations per 100,000 population using marginal effects, by county presence of at least one mAb treatment site

|  | **COVID-19 Hospitalization Rate (per 100k population)** | | |
| --- | --- | --- | --- |
| **N** | 2580 |  |  |
| **Clusters** | 47 |  |  |
|  | **Counties with mAb treatment site(s)** | **Counties without mAb treatment site(s)** | **Difference (between group, pre-post)** |
| **Weeks Since July 1, 2021** |  |  |  |
| Nov 2020 – June 2021 | 9.33 (2.41) | 12.27 (3.08) | – |
| 1 | – | – |  |
| 2 | – | – |  |
| 3 | 4.23 (0.95) | 6.98 (2.45) | 0.19 |
| 4 | 3.68 (0.79) | 8.80 (3.01) | -2.18** |
| 5 | 4.50 (1.00) | 9.31 (2.99) | -1.87** |
| 6 | 3.63 (0.59) | 7.73 (2.04) | -1.16** |
| 7 | 4.32 (0.74) | 8.31 (2.17) | -1.05* |
| 8 | 5.06 (0.87) | 7.95 (2.13) | 0.05 |
| 9 | 5.57 (1.11) | 8.02 (1.95) | 0.49 |
| 10 | 6.27 (1.06) | 7.90 (1.80) | 1.30 |
| 11 | 5.91 (1.07) | 6.74 (1.71) | 2.11 |
| 12 | 5.84 (0.95) | 6.78 (1.52) | 2.00 |
| 13 | 6.40 (1.08) | 6.85 (1.40) | 2.49 |
| 14 | 6.27 (1.06) | 7.85 (1.71) | 1.35 |
| 15 | 7.25 (1.30) | 7.49 (1.48) | 2.70 |
| 16 | 6.94 (1.11) | 7.62 (1.53) | 2.25 |
| 17 | 7.60 (1.32) | 8.87 (1.62) | 1.67 |
| 18 | 8.31 (1.40) | 8.47 (1.90) | 2.77 |
| 19 | 9.30 (1.55) | 8.99 (2.06) | 3.25* |
| 20 | 9.02 (1.62) | 8.86 (2.19) | 3.11 |
| 21 | 9.26 (1.62) | 8.53 (2.04) | 3.66* |
| 22 | 9.17 (1.57) | 9.93 (2.27) | 2.17 |
| 23 | 8.22 (1.47) | 8.71 (2.06) | 2.44 |
| 24 | 7.30 (1.28) | 7.96 (2.15) | 2.27 |
| 25 | 5.91 (1.15) | 5.83 (1.97) | 3.02 |
| 26 | 5.06 (1.20) | 5.29 (1.91) | 2.70 |

*Note.* Results displayed as marginal effects (incidence rates). The standard errors are clustered at the county-level. Robust standard errors are in parentheses. **p*<0.1; ***p*<0.05; ****p*<0.001
